# The Influence of Hip-Strengthening Program on Patients with Chronic Ankle Instability

**DOI:** 10.3390/medicina60081199

**Published:** 2024-07-24

**Authors:** Woo-Jin Yeum, Mi-Young Lee, Byoung-Hee Lee

**Affiliations:** 1 Graduate School of Physical Therapy, Sahmyook University, Seoul 01795, Republic of Korea; 2 Department of Physical Therapy, Sahmyook University, Seoul 01795, Republic of Korea; mylee@syu.ac.kr

**Keywords:** hip joint, strength, balance, function

## Abstract

*Background and Objectives*: Repetitive ankle sprains lead to mechanical instability of the ankle. Patients with chronic ankle instability may experience decreased muscle strength and limited postural control. This study investigated the effects of a hip-strengthening exercise program on muscle strength, balance, and function in patients with chronic ankle instability. *Materials and Methods*: A total of 30 patients participated in the study and were randomly assigned to the two groups. Among the 30 participants, 14 were assigned to the hip joint-strengthening exercise group and 16 to the control group. The experimental group underwent a hip-strengthening exercise program and received training for 40 min per session twice a week for four weeks. The control group received the same frequency, duration, and number of sessions. Measurements were performed before and after the training period to assess changes in hip strength, balance, and function. *Results*: In the within-group and between-group comparisons, both groups showed significant differences in hip joint strength, static balance, dynamic balance, and function (FAAM; foot and ankle ability measures) (*p* < 0.05). Statistically significant differences were observed in the time × group interaction effects among the hip abductors and external rotation in hip joint strength, path length in static balance, posterolateral and posteromedial in dynamic balance, and FAAM-ADL and FAAM-SPORT functions (*p* < 0.05). *Conclusions*: Accordingly, this study confirmed that hip joint-strengthening exercises have a positive effect on the strength, balance, and function of patients with chronic ankle instability, and we believe that hip joint-strengthening exercises will be recommended as an effective intervention method for patients suffering from chronic ankle instability.

## 1. Introduction

Ankles are the most common site of injury and damage in 70 different sports [1]. The most commonly injured ligament during a lateral ankle sprain is the anterior talofibular ligament; simultaneous injury to the calcaneofibular ligament creates a much larger injury [2]. Typically, the treatment for acute lateral ankle sprains varies according to the grade. Grades I and II usually involve conservative treatments such as bracing, taping, functional treatment, and physical therapy. Grade III, indicating a complete tear, often requires surgical intervention [3]. Approximately 70% of ankle injuries recur, and approximately 30% of lateral ankle sprains (LASs) lead to recurrent instability, which is termed chronic ankle instability (CAI) [4]. Individuals with chronic ankle instability typically experience ankle instability that persists for >12 months after the initial injury, leading to limitations in participating in daily activities. This instability is characterized by damage to the supporting structures of the ankle and can result in various mechanical and functional instabilities [5]. The characteristics of individuals with chronic ankle instability may include decreased muscle strength and proprioception, as well as limitations in joint range of motion. These factors can lead to compensatory movements involving proximal muscles such as the hip abductors when attempting to maintain balance on the affected limb, causing alterations in central proprioception [6]. Balance and postural control involve the integration of sensory inputs, such as proprioception and vestibular sensation, to maintain static and dynamic balance against gravity. Posture control is associated with changes in proximal stability and centripetal neural control, as well as proprioceptive perception [7,8]. Acute ankle sprains can damage the joint mechanoreceptors and centripetal nerves, thereby contributing to proprioceptive deficits in patients with chronic ankle instability. Compared to healthy individuals, there are deficiencies in proprioception and joint position sense during inversion and eversion movements of the ankle [9].

Proprioceptive training includes exercises such as balancing on one leg with the eyes closed, balancing on a wobble board or disk, and activities involving catching or throwing a ball. When applied long-term, exercises like these have proven highly effective for patients with chronic ankle instability, and short-term applications have shown effectiveness as well [10]. Balance training has been shown to improve function, instability, and dynamic balance in patients with chronic ankle instability, as well as self-reported function and quality of life [11]. Weakness of the hip muscles is a risk factor for ankle sprains, and increased hip movement in the sagittal and frontal planes during walking leads to greater use of hip strategies [12].

Maintaining balance at the stance phase during walking is crucial, and the coordination between the hip and tibiofibular joint controls foot placement and the center of mass, playing a significant role in ankle function [13]. Weakness of the hip abductor muscles, such as the gluteus medius, is associated with injuries to the lateral ankle ligaments and ankle instability. Patients with chronic ankle instability have been found to exhibit reduced activity in muscles around the ankle, knee, and hip during typical functional rehabilitation exercises [14].

Patients with chronic ankle instability typically exhibit reduced bilateral activation of the hip abductor muscles during most instances of walking compared with healthy individuals, along with weakness in the hip muscles [15,16]. Increasing hip muscle strength can help prevent future ankle instability [17,18]. Hip-strengthening exercises improve pain and joint function in patients with chronic ankle instability, whereas resistance band exercises enhance hip muscle activation and proprioceptive sensation.

Resistance training helps reduce the risk of physical and functional limitations through strength gains and is utilized for injury prevention and rehabilitation in sports activities [19,20,21]. Research on balance training interventions for patients with chronic ankle instability has consistently been reported; however, studies on hip muscle-strengthening exercises are lacking. Therefore, this study aimed to investigate the effects of a hip strength training program on muscle strength, balance, and function in patients with chronic ankle instability.

## 2. Materials and Methods

### 2.1. Subjects

Before recruiting participants for this study, we performed a power analysis using G*Power version 3.1.9.7 (Heinrich-Heine Universität, Düsseldorf, Germany). ANOVA: repeated measures; within–between interaction; an effect size f of 0.25 was obtained for all of the outcome measures, with an α error probability of 0.05, to minimize type 1-β errors; probability of 80%; number of groups of 2; and number of measurements of 4. As the estimated target sample size was 32, we recruited 45 participants who underwent physical therapy. This study consisted of 32 patients, including 16 males and 16 females, and was conducted with non-athlete participants. Information on physical activity levels was not collected.

This study is a randomized controlled trial (RCT) comparing the pre- and post-experiment results of two groups, and the selection criteria for research participants adhered to the International Ankle Consortium guidelines and included individuals with at least one significant history of ankle sprain and, in cases of acute sprains, individuals whose injury occurred at least 12 months prior to study registration.

Individuals with inflammatory symptoms (pain, swelling, etc.), those who have experienced a disruption of desired physical activity for more than one day due to instability, individuals who have experienced “giving way” at least twice within one year, and those with Cumberland Ankle Instability Tool (CAIT) scores of 24 or lower were selected as participants [22].

Exclusion criteria, according to the International Ankle Consortium, included individuals with a history of previous surgery on the musculoskeletal structures (i.e., bones, joint structures, and nerves) of both lower limbs, individuals with a history of fractures in both lower limbs requiring realignment, and individuals who have experienced a disruption of desired physical activity for at least one day due to acute injury to the musculoskeletal structures of other joints in the lower limbs within the previous 3 months, affecting the integrity and function of the joint (e.g., sprains, fractures) [22].

All participants were briefed on the purpose and details of the study and guided on the procedure to withdraw from participation at any time without repercussions. Written informed consent was obtained and submitted. All participants signed a consent form after the procedure and the purpose of the study were explained. This study was approved by the Sahmyook University Institutional Review Board (approval number: SYU 2023-04-004-001) and Clinical Research Information Service (KCT0008990). Participants fully understood the objectives and procedures used in the study. This study adhered to the ethical principles of the Declaration of Helsinki.

### 2.2. Experimental Procedure

This study collected information on the clinical characteristics of the research participants, such as medical history and present conditions, as well as general characteristics, including age, height, weight, and body mass index (BMI), after obtaining approval to access medical records prior to the experiment. The height and weight of the participants were measured using a stadiometer and scale (BIB-BSM 370; InBody, Seoul, Republic of Korea, 2009) located in the clinic.

To minimize bias and errors related to the experiment during group selection, the Research Randomizer program (http://www.randomizer.org/, accessed on 24 May 2023) was used to randomly allocate the participants into two groups. The assignment was then administered to therapists to further minimize bias, thereby facilitating the experiment.

Both groups underwent pre- and post-experimental evaluations, including assessment of hip flexion, extension, and external rotation strength and static and dynamic balance abilities and functional evaluations using self-reported functional questionnaires. This study recruited 32 participants, but 2 participants in the experimental group dropped out due to health-related issues, resulting in a final statistical analysis with 30 participants.

Statistical analysis was conducted on 14 participants in the experimental group and 16 participants in the control group after excluding 2 dropouts from the experimental group during the post-test evaluations.

In the experimental group, hip-strengthening exercises were performed twice a week for a total of eight sessions over 4 weeks, with each session lasting 10 min. Dynamic balance exercises were applied twice a week for a total of eight sessions over 4 weeks, with each session lasting 20 min. Additionally, conventional physiotherapy treatments conducted in the hospital, such as heat therapy and interferential current therapy, were applied twice a week for a total of eight sessions over 4 weeks, with each session lasting 10 min. In the control group, dynamic balance exercises were applied twice a week for a total of eight sessions over 4 weeks, with each session lasting 30 min. Conventional physiotherapy was also applied twice a week for a total of eight sessions over 4 weeks, with each session lasting 10 min. The pre- and post-evaluations were conducted by physical therapists with more than three years of experience at the same location. Additionally, to ensure the safety of the participants during assessments and experiments, a physical therapist with over three years of experience was present throughout the research process to provide continuous assistance.

The therapists responsible for the interventions underwent one week of training on the safety of the experiment and familiarized themselves with the proper use of measurement equipment before the start of pre-education and practice evaluations. This was performed to minimize errors and ensure the safety and accuracy of the experiments. Both the experimental and control groups were provided with treatments and environments under identical conditions. After the experiment, the same strength measurements, balance assessments, and functional evaluations were repeated as during the pre-test. The results were compiled and statistically analyzed.

### 2.3. Training Program

#### 2.3.1. Hip-Strengthening Exercises

The experimental method involved selecting the position where the external rotator and abductor muscles exerted maximal voluntary isometric contractions (MVIC%) based on previous studies. Additionally, the experiment was reconstructed with reference to previous studies [23].

Resistance bands were used for hip-strengthening exercises in this study. Resistance bands effectively increase muscle strength, aid muscle strengthening, and enhance physical activity [24]. In this study, the resistance bands were arranged in the following order according to their resistance levels: yellow, green, and blue. During the first two weeks, a yellow band was used. The ankles of the participants were strapped with resistance bands of varying intensities to create weekly difficulty levels. Participants were allowed to choose the color of the resistance band based on the level of resistance that they could handle.

The experiment was conducted on participants who complained of chronic ankle instability on both sides because experiencing instability on both sides tended to induce a greater sense of instability. The exercise for the hip external rotator muscles involved having the subject lie on their side without bending the knees and then externally rotating the hip to approximately 30° to maximize the contraction of the gluteus medius muscle [21]. Exercises for the hip external rotator muscles were performed for 12–15 repetitions per set for a total of three sets. The rest time between sets was 30 s. For the hip flexor exercise, the participants stood facing a wall with resistance bands tied around their ankles. Without bending their knees, the participants flexed their hip joints by approximately 20°.

Hip flexor exercises were performed for 12–15 repetitions per set, for a total of three sets. The rest time between sets was 30 s. For the hip external rotation exercise, the participants sat with resistance bands tied around their ankles and bent their knees to a 90-degree angle. The participants then performed an external hip rotation of approximately 45°. To prevent unnecessary movements, the participants held both knees with their hands to stabilize their torso. The hip external rotation exercise was performed for 12–15 repetitions per set for a total of three sets. The rest time between sets was 30 s (Table 1).

#### 2.3.2. Dynamic Balance Exercises

The experimental method for dynamic balance exercises was reconstructed based on previous studies [25], and the difficulty level was set according to the equipment used, progressing from a foam mat (Balance Pad Elite; Airex^®^, London, UK, 2020) to an air cushion (Dynair^®^ XXL Meditation; TOGU©, Rosenheim, Germany, 1996) and finally to a BOSU^®^ balance trainer (BOSU^®^ NexGen™ Home Balance Trainer; BOSU^®^, Newport Beach, CA, USA, 1999). The intensity was adjusted by allowing or blocking visual feedback. In a study by Strom et al. [26], the BOSU^®^ balance trainer exhibited more than twice the amount of kinematic variability compared to the foam mat. Additionally, Cheatham et al. [27] categorized the BOSU balance trainer as being at the fourth level of difficulty for both two-legged and single-legged supports on an unstable surface in healthy adults. The air-filled disc, similar in shape to the air cushion, was categorized as Level 3 difficulty, whereas the foam pad, resembling the foam mat, was considered Level 2 difficulty, indicating a relatively easier level. Furthermore, in weeks 1–2, the difficulty of instability increased from two-legged to single-legged support, and from week 3 onwards, functional movements such as squats were included. At week 4, exercises aimed at increasing dynamic balance, such as single-leg swings, ball passing, and single-leg squats, were performed (Table 2).

#### 2.3.3. Outcome Measures

##### Muscle Strength

To measure the muscle strength of the hip joint, we used the Lafayette Manual Muscle Testing System (Model 01163; Lafayette Instrument Company, Lafayette, IN, USA, 2020) to measure the maximum muscle strength of hip joint abduction, flexion, and external rotation. The method of measuring hip joint external rotation involved the subject lying supine with the hip joint maintained in a neutral position. The leg was tested, and the resistance point was positioned at the edge of the table. The opposite leg was bent, and the subject held both hands on the side of the table. Resistance was applied for 3 s at a fixed position, with the resistance point placed 5 cm proximal to the lateral edge of the greater trochanter at the level of the muscle belly. Hip joint flexion was measured with the subject lying prone, with the hip joint maintained in a neutral position while holding onto the side of the table with both hands. Resistance was applied for 3 s at a fixed position, with the resistance point positioned 5 cm proximal to the medial edge of the lesser trochanter on the posterior aspect of the thigh.

The method for measuring hip joint external rotation involved assuming a seated position with the hip joint flexed at 90° while holding it onto the side of the table with both hands. The researcher applied resistance at a fixed position, with the resistance point positioned 5 cm proximal to the medial edge of the lesser trochanter for external hip joint rotation [28].

The Lafayette Digital Muscle Tester (Model 01163, Lafayette Instrument Company, Lafayette, IN, USA, 2020) features a lightweight (10.6 oz) microprocessor-controlled device that measures the maximum force (in pounds or kilograms), the time to reach the maximum force, and the total test duration. The range of test time is from 1 to 10 s, and a signal sound is emitted when the preset time is reached [29]. The reliability of the hip joint muscle strength measurement was ICC = 0.92, and the measurement time was set to 3 s [30].

##### Static Balance Ability

A force plate (PDM Multifunction Force Measuring Plate; Zebris, Kaufbeuren, Germany, 2004) was used to measure static balance ability. The force plate consisted of a 32 cm × 47 cm platform with 1504 pressure sensors (force sensors) installed, one per square centimeter, to measure static pressure on the feet during standing or walking. Each sensor was independently measured in the pressure measurement range of 1–120 N/cm^2^. The static sample pressure extraction rate ranged from 2 to 5 Hz, the dynamic sample pressure extraction rate was approximately 90 Hz, and the accuracy was ±5%. Furthermore, the reliability of the force plate is ICC = 0.83–0.99 [31]. The measurements were repeated three times, and the mean values were calculated using the confidence ellipse area (CEA), path length (PL), and average velocity (AV).

The subjects stood on a force plate with both legs held onto the center point and baseline. Upon the signal “start” from the measurer, the leg on the supporting side remained relaxed, while the leg on the opposite side was slightly bent and raised. This position was held for 30 s, and upon the signal “stop”, the raised leg was lowered. The participants performed a total of three experiments, and the average values were recorded for statistical analysis. The measurements were conducted with the eyes open and closed. During measurements with eyes closed, one assistant remained on standby at all times to ensure the safety of the subject.

Dynamic balance was assessed using the Y-Balance Test. In a study focusing on subjects with chronic ankle instability, positive correlations were found between hip abduction strength, hip flexion strength, and posterior–medial and posterior–lateral reach distances in the Star Excursion Balance Test (SEBT) [32]. The Y-Balance Test involves measurements in three directions: posteromedial, posterolateral, and anterior. With the anterior direction as the reference, the posteromedial and posteromedial directions form a 135-degree angle, and the angle between the posteromedial and posteromedial directions is 90°. The intermediate ICC values for inter-rater reliability were as follows: for the anterior direction, ICC = 0.88 (range = 0.83~0.96); for the posterior–medial direction, ICC = 0.87 (range = 0.80~1.00); and for the posterior–lateral direction, ICC = 0.88 (range = 0.73~1.00). The intermediate ICC values for intra-rater reliability were as follows: for the anterior direction, ICC = 0.88 (range = 0.84~0.93); for the posterior–medial direction, ICC = 0.88 (range = 0.85~0.94); and for the posterior–lateral direction, ICC = 0.90 (range = 0.68~0.94) [33].

The participant placed the supporting leg on the starting block and extended the experimental leg as far as possible in the anterior, posterolateral, and posteromedial directions. The scores for each direction (anterior, posterior–lateral, and posterior–medial) were calculated by dividing the average reach distance (cm) by the participant’s leg length (cm) and multiplying by 100 to obtain the percentage of leg length [34].

##### Function

The FAAM is a self-reported assessment method developed to comprehensively evaluate the physical performance of individuals with various foot and ankle disorders. This functional scale comprises two subscales: activities of daily living (ADL; 21 items) and sports (8 items) [35]. A 5-point Likert scale, ranging from 0 (not difficult at all) to 4 (unable to do), was utilized with an additional option of “not applicable” to evaluate each item. The total score for each subscale was converted to a scale from 0 (maximum disability) to 100 (minimum disability). Scores were then complemented by a global 0–100 rating for each subscale and a comprehensive 4-point Likert scale for overall function [35].

The FAAM is a valid and reliable assessment tool demonstrating validity and reliability for patients with a wide range of musculoskeletal disorders affecting the lower limbs, feet, and ankles [36]. For FAAM-ADL and SPORT, the ICCs were 0.89 and 0.87, respectively. The minimum detectable change, based on a 95% confidence interval, was ±5.7 points for the ADL subscale and ±12.3 points for the sports subscale [37].

#### 2.3.4. Data Analysis

All statistical analyses were performed using SPSS version 23.0 for Windows software (SPSS Inc., Chicago, IL, USA). The mean and standard deviation were calculated for data analysis. The general characteristics of the subjects were examined for normal distribution using the Shapiro–Wilk test. Furthermore, an independent *t*-test was conducted to assess the homogeneity between the groups. Within-group pre- and post-intervention differences were analyzed using paired *t*-tests. Between-group differences were compared using independent *t*-tests. Additionally, repeated-measures analysis of variance (ANOVA) was used to evaluate the interaction effect between groups and time. The significance level for all statistical analyses was set at *p* < 0.05.

## 3. Results

### 3.1. General Characteristics of Participants

This study included 14 and 16 participants in the HSE and control groups, respectively. In this study, all general characteristics and dependent variables were normally distributed, and the homogeneity test showed no significant differences between the groups for any of the dependent variables in the pre-assessment results (Table 3).

### 3.2. Changes in Hip Joint Strength

When examining the changes in hip muscle strength following hip-strengthening exercises, both the experimental and control groups demonstrated significant post-intervention improvements (*p* < 0.05), and the experimental group showed significant differences in hip extensors, hip abductors, and external rotation compared to the control group (*p* < 0.001).

In terms of the time effect, there were significant differences in the hip extensors, hip abductors, and external rotation (*p* < 0.001). Regarding the group effect, the hip extensors showed significant differences (*p* < 0.01). Additionally, significant time × group interaction effects were observed in the hip abductors and external rotation (*p* < 0.001) (Table 4).

### 3.3. Changes in Static Balance

When examining the changes in static balance following hip-strengthening exercises, both the experimental and control groups demonstrated significant post-intervention improvements (*p* < 0.001). In the comparison of balance between groups, the experimental group showed significant differences compared to the control group PL (*p* < 0.001).

In terms of the time effect, significant differences were observed in CEA, PL, and AV levels (*p* < 0.001). Additionally, significant time × group interaction effects were observed for PL (path length) (*p* < 0.01) (Table 5).

### 3.4. Changes in Dynamic Balance

The Y-Balance Test was used to assess dynamic balance ability. When examining the changes in dynamic balance following hip-strengthening exercises, both the experimental and control groups demonstrated significant improvements post-intervention in the anterior, posterior–lateral, and posterior–medial directions (*p* < 0.001). In the comparison of balance between groups, the experimental group showed significant differences compared to the control group in the posterior–lateral and posterior–medial directions (*p* < 0.001). In terms of the time effect, significant differences were observed in the anterior, posterolateral, and posteromedial directions (*p* < 0.001). Regarding the effect of the group, the posterolateral direction was significant (*p* < 0.05). Additionally, significant time × group interaction effects were observed in the posterolateral and posteromedial directions (*p* < 0.001) (Table 6).

### 3.5. Changes in Foot and Ankle Ability Measure

The FAAM-ADL/SPORT was used to assess self-reported functional measurements. Following hip-strengthening exercises, changes in function were examined, and both the experimental and control groups demonstrated significant improvements post-intervention in FAAM-ADL and FAAM-SPORT (*p* < 0.001). In the comparison of function between the groups, the experimental group showed significant differences compared to the control group in FAAM-ADL and FAAM-SPORT scores (*p* < 0.01).

In terms of the time effect, there were significant differences between the FAAM-ADL and FAAM-SPORT groups (*p* < 0.001). Additionally, significant time × group interaction effects were observed for FAAM-ADL and FAAM-SPORT (*p* < 0.01) (Table 7).

## 4. Discussion

This study aimed to evaluate the impact of hip-strengthening exercises on muscle strength, balance, and function in patients with chronic ankle instability. The results confirmed that these exercises effectively improve hip strength, balance, and functional performance. Consequently, hip-strengthening exercises should be considered an effective treatment option for chronic ankle instability. Future research should explore the benefits of balance training with visual feedback.

### 4.1. Changes in Hip Joint Strength

Hip joint strength is important for maintaining ankle stability, and injuries to the lower extremities can result in decreased dynamic postural control of the lumbopelvic complex [18]. The hip abductor muscles are crucial for controlling and stabilizing the pelvis during movement. They exhibit the greatest muscle activity during the mid-to-late stance phase of walking, and the strength of the gluteal muscles is highly important during activities such as stair climbing and functional tasks [38]. Patients with chronic ankle instability often have insufficient hip external rotator strength, and hip muscle weakness contributes to decreased dynamic balance. Better hip flexor muscle strength correlates with improved dynamic stability [39].

The greatest influence on ankle sprains was attributed to the gluteus maximus (r = 0.90), followed by the gluteus medius (r = 0.49). Furthermore, a strong correlation was reported between hip muscle strength (*p* = 0.011) and ankle sprains (*p* = 0.002) [40]. Weakening of hip external rotation and abduction strength can increase knee varus torque. An increase in the Q-angle of the knee can overactivate the hip external rotator muscles during walking or movement, potentially leading to other lower-limb injuries [41]. Furthermore, weakness of the hip external rotator muscles leads to persistent lateral ankle sprains in adolescent soccer players. Additionally, regardless of physical characteristics, hip flexor muscle weakness is considered a risk factor for ankle sprains [42].

In the comparison of muscle strength between the groups, the experimental group showed significant differences in hip extensors, hip abductors, and external rotation compared to the control group (*p* < 0.001). Additionally, significant time × group interaction effects were observed in the hip abductors and external rotation (*p* < 0.001).

This suggests that hip flexion exercises performed in a supine position during hip-strengthening exercises may strengthen weakened gluteus maximus, quadriceps, hamstrings, and calf muscles [43]. Consequently, it is speculated that an increase in the functional activity of the hip joint due to balance training programs may have occurred [33]. Additionally, it is conjectured that during hip-strengthening exercises using resistance bands, contraction of the posterolateral fibers of the gluteus medius may increase muscle strength, thereby altering hip kinematics [21,23].

This study focused on exercises involving hip flexion, extension, and external rotation, with an emphasis on the gluteus maximus and medius. External hip rotation exercises have been found to activate the posterolateral fibers of the gluteus maximus and medius [44]. Both the experimental and control groups showed significant improvements in overall hip muscle strength parameters in this study. However, the experimental group, which focused on hip-strengthening exercises, demonstrated a greater increase than the control group. The reason for the increase in muscle strength in the control group, which did not perform hip-strengthening exercises, is likely that the participants used hip strategies to maintain balance instead of relying on their unstable ankles during the balance training program, resulting in an improvement in the gluteus medius. Additionally, blocking visual feedback and exercising on an unstable surface would have activated not only the gluteus medius but also the somatosensory system. Consequently, there was a significant difference in muscle strength improvement between the hip-strengthening exercise group and the control group (*p* < 0.001), and a significant interaction effect between the experimental and control groups was observed in hip abduction and external rotation (*p* < 0.001). This suggests a strong correlation between hip strength and ankle instability, and it is believed that the direct strengthening exercise intervention significantly improved the muscle strength increase in the experimental group.

### 4.2. Changes in Static Balance Ability

Patients with chronic ankle instability often lack the ability to detect joint position and movement in previously sprained ankles, resulting in impaired neuromuscular response balance associated with proprioceptive deficits related to damage to mechanoreceptors in the ankle joint and surrounding tissues. Peripheral sensory deficits such as instability and laxity are linked to functional and structural changes in the brain, leading to subsequent impairments in proprioception [45]. Such impairments affect static balance abilities, and general balance training consisting of functional exercises and single-leg balance exercises over a period of four weeks can effectively enhance self-reported function and dynamic and static postural control during physical activities [46].

In this study, there were significant differences in CEA, PL, and AV levels over time (*p* < 0.001). Additionally, there was a significant time × group interaction effect in the PL (*p* < 0.01). This suggests that balance training increased cortical spinal excitability and spinal reflex control ability, and through hip-strengthening exercises, postural stability increased, leading to a positive impact on PL in the experimental group compared to the control group [47].

Furthermore, this study aimed to enhance ankle control by creating stability in the peroneal muscles through compensatory postural adjustments using visual feedback and hip-strengthening exercises on an unstable surface. Both the experimental and control groups underwent balance training with the eyes open and closed. However, the short-term hip strengthening created by four weeks of training did not provide enough control ability to replace balance ability in the condition of visual blockage. This is similar to Song’s study [48], which reported no difference in static balance ability between the eyes-open and eyes-closed conditions in patients with chronic ankle instability while maintaining a single-leg stance; as both the experimental and control groups performed the same balance exercises under similar conditions, it was presumed that both groups would have experienced a similar increase in balance ability.

### 4.3. Changes in Dynamic Balance Ability

Dynamic balance refers to the ability to maintain trunk stability and resist force during daily and sports activities. Muscles such as the gluteus maximus and medius of the hip contribute to stability [49]. The Y-Balance Test, a valid and reliable tool for assessing dynamic balance ability, not only evaluates balance but also predicts lower extremity injuries, such as chronic ankle instability [50]. The Y-Balance Test showed significant correlations with hip abduction, adduction, and external rotation, indicating that weaker hip abduction strength is correlated with lower Y-Balance Test scores [34].

Cain et al. [51] conducted a study applying balance training combined with resistance exercises for four weeks. Their results showed an increase from 76.64% to 79.43% in the anterior direction, which did not reach the minimal detectable change (MDC) value of 6.5%. In this study, the experimental group also showed a significant improvement in the anterior direction, from 65.83% before the exercise to 68.81% after the exercise (*p* < 0.001), while the control group showed a significant improvement from 63.26% before the exercise to 65.59% after the exercise (*p* < 0.001). However, the increase before and after the intervention was minimal compared to other directions. This is because, unlike the posterior–lateral and posterior–medial directions, which involve hip abduction, extension, and external rotation by tilting the torso forward and moving the legs backward, the anterior direction requires hip flexion strength to counteract the hip extensors [16,17]. This study focused on concentric and isometric contractions, and the lack of eccentric contraction strength of the hip extensors to counteract the hip flexion strength might have prevented a significant difference between the two groups in the anterior direction. Consequently, there was no significant difference in group effects and time × group interactions.

In this study, a significant group effect was observed in the posterolateral direction (*p* < 0.05). The experimental group showed a significant improvement from 81.45% before exercise to 92.10% after exercise (*p* < 0.001), whereas the control group showed a significant improvement from 79.76% before exercise to 85.94% after exercise (*p* < 0.001).

In a study by Wright et al. [52], chronic ankle instability patients with CAI were divided into a resistance exercise group and a wobble board balance exercise group for four weeks. The results showed that in the posterior–medial direction of the Y-balance Test, the wobble board balance exercise group increased their score from 0.98 ± 0.09 before intervention to 1.03 ± 0.08 after intervention (*p* < 0.001), while the resistance exercise group increased their score significantly from 0.92 ± 0.1 before the intervention to 1.00 ± 0.08 after the intervention (*p* < 0.001) for both groups.

In this study, we presumed that concurrent balance and resistance exercises on an unstable surface would lead to significant improvements in both posteromedial and posterolateral directions.

### 4.4. Changes in Self-Reported Function

The self-reported function perceived by patients with chronic ankle instability is an essential function that diminishes the likelihood of persistent instability and recurrent ankle sprains, determining the return to daily and sports activities. Approximately 72% of chronic ankle instability patients fail to regain their previous level of physical activity, resulting in decreased function and quality of life [53]. Moreover, patients with chronic ankle instability experience diminished self-reported function due to fear of reinjury during daily or sports activities. FAAM-ADL and -SPORT are appropriate tools for evaluating the physical function of individuals with ankle injuries and quantifying functional impairment in patients with chronic ankle instability based on subjective information [54]. In particular, there is a moderate correlation between hip abductor strength and FAAM [55].

In Cain et al.’s [51] study, a 4-week ankle rehabilitation program combining resistance exercise and balance training using a BAPS board in adolescent athletes with chronic ankle instability resulted in a significant increase in FAAM-ADL scores from pre-intervention, at 85.36 ± 11.42, to post-intervention, at 91.31 ± 8.80 (*p* < 0.001); FAAM-Sports scores increased significantly from pre-intervention, at 74.06 ± 14.44, to post-intervention, at 84.69 ± 17.21 (*p* < 0.001).

In this study, FAMM-ADL showed a significant improvement in the experimental group, increasing from 77.99% before exercise to 89.61% after exercise (*p* < 0.001). This suggests that patients with chronic ankle instability experience significantly reduced activity of the peroneal muscles from initial contact to the terminal stance during walking [56], and activating the peroneal muscles through hip-strengthening exercises resulted in the observed improvement. Furthermore, in this study, FAAM-ADL showed a significant effect over time (*p* < 0.001), and there were significant differences between the groups and a significant time × group interaction effect (*p* < 0.01). This improvement is presumed to have occurred because of increased hip muscle strength in daily life activities such as “walking for a long time” and “walking on an unstable surface.” Additionally, because the age group consisted of individuals aged 20–39, a relatively young age group, it can be inferred that even short-term interventions are effective in reducing instability.

In this study, FAMM-SPORT showed a significant improvement from 64.73% before exercise to 76.78% after exercise in the experimental group (*p* < 0.001). This improvement may be attributed to tasks in the FAAM-Sports section such as “running”, “jumping”, and “landing”, which rely on hip muscle function. Hip and knee lower limb strength may have contributed to stability during movements like vertical jumps and landings, thereby reducing instability, as suggested by Cain et al. and Smith et al. [4,51].

Anguish and Sandrey [56] demonstrated significant time effects for both FAAM-ADL and -SPORT after four weeks of two types of balance training in patients with chronic ankle instability (*p* < 0.001). Wright et al. [52] compared wobble board balance training and resistance band exercises in patients with chronic ankle instability and observed significant time-by-group interactions for FAAM-ADL (*p* < 0.001). Both FAAM-ADL and -SPORT showed significant time effects (*p* < 0.001), consistent with the findings of this study. The contrasting results in the self-reported functional outcome measures may arise from the subjective nature of the assessment items in the questionnaire, leading to significant variations depending on individual quality of life or environmental factors. Furthermore, it is speculated that there may be differences in performance on the FAAM-Sports questionnaire based on individual physical abilities, even when the same intervention was administered.

A limitation of this study was the lack of outpatient participants owing to the nature of the hospital, resulting in an insufficient sample size for analyzing differences based on sex, bilateral versus unilateral conditions, and setting up a coper group. In future research, analyzing a larger number of participants and subgroups based on these factors could allow for the generalization of the research findings. Second, owing to the situation in Korean hospitals, there was a lack of intervention frequency and time, which prevented long-term follow-up observations, thus hindering the confirmation of long-term effects. Third, owing to the simplification of the intervention method, only resistance exercises were performed among the various hip-strengthening exercises, leading to a limited variety of exercises. Therefore, future research incorporating various functional strengthening exercises may demonstrate a greater effect size. Fourth, dynamic balance measurements using force plates, electromyography during walking, or assessment of walking patterns were limited post-intervention. Chronic ankle instability patients with CAI are influenced by muscle activation from initial contact to the terminal stance during walking due to weakened hip muscle strength. Future studies should include these measurements to provide a comprehensive understanding of the effects of the intervention. Therefore, future studies could accurately measure the differences in walking variables due to hip muscle strength enhancement by incorporating various measurements, such as walking variables and force plate assessments. Additionally, structural characteristics, such as foot length and width, and the Foot Posture Index should be considered.

## 5. Conclusions

This study investigated the effects of hip-strengthening exercises on muscle strength, balance, and function in patients with chronic ankle instability. This study confirmed that hip-strengthening exercises are effective interventions for improving hip muscle strength, static balance, dynamic balance, and self-reported function, suggesting that hip-strengthening exercises can be proposed as an effective intervention method for patients with chronic ankle instability. Future studies should consider the effects of balance training with visual feedback on the balance ability of patients with chronic ankle instability. Incorporating hip-strengthening exercises into rehabilitation programs for chronic patients is also recommended.

## Figures and Tables

**Table 1:** Hip-strengthening exercises.

Week	Hip-Strengthening Exercises	Time
Week 1~2	1. Side-lying hip external rotation (yellow) 2. Standing hip flexion (yellow) 3. Seated hip external rotation (yellow)	Each exercise: 12–15 repetitions/3 sets
Week 3	1. Side-lying hip external rotation (green) 2. Standing hip flexion (green) 3. Seated hip external rotation (green)	Each exercise: 12–15 repetitions/3 sets
Week 4	1. Side-lying hip external rotation (blue) 2. Standing hip flexion (blue) 3. Seated hip external rotation (blue)	Each exercise: 12–15 repetitions/3 sets

The total exercise duration was 30 min, with a rest period of 30 s between sets.

**Table 2:** Dynamic balance exercises.

Week	Dynamic Balance Exercises	Time
Week 1	1. Balancing on BOSU ball with both legs (eyes closed) 2. Balancing on foam mat with one leg (eyes closed) 3. Balancing on foam mat with one leg (eyes open)	30 s to 60 s 3 to 5 repetitions Total of 3 sets
Week 2	1. Balancing on air cushion with both legs (eyes open) 2. Balancing on air cushion with both legs (eyes closed) 3. Balancing on BOSU ball with one leg (eyes open) 4. Balancing on BOSU ball with one leg (eyes closed)	30 s to 60 s 3 to 5 repetitions Total of 3 sets
Week 3	1. Balancing on air cushion with one leg (eyes open) 2. Balancing on air cushion with one leg (eyes closed) 3. Squatting on BOSU ball (eyes open) 4. Squatting on BOSU ball (eyes closed)	30 s to 60 s 3 to 5 repetitions 10 to 15 repetitions Total of 3 sets
Week 4	1. Passing ball on BOSU ball 2. Single-leg swings on BOSU ball 3. Single-leg squats on BOSU ball (30–45°)	10 to 15 repetitions Total of 3 sets

The total exercise time was 30 min, and the rest time between sets was set to 30 s.

**Table 3:** General characteristics of participants (*n* = 30).

Characteristics	HSE Group (*n* = 14)	Control Group (*n* = 16)	*t* (*p*)
Age (years)	27.86 (4.16) ^a^	27.63 (3.57)	−0.164 (0.871)
Height (cm)	167.93 (6.92)	171.19 (9.19)	1.084 (0.288)
Weight (kg)	64.86 (14.21)	66.50 (16.23)	0.293 (0.772)
BMI (kg/m^2^)	22.88 (3.85)	21.80 (4.18)	−0.730 (0.472)

^a^ M(SD); BMI = body mass index; HSE group = Hip-strengthening exercise group.

**Table 4:** Pre- and post-intervention hip joint muscle strength in hip strength exercise group and controls.

Parameters		HSE Group (*n* = 14)	Control Group (*n* = 16)	*t* (*p*)	Time *F* (*p*)	Group *F* (*p*)	Time × Group *F* (*p*)
Hip extension (*N*)	Pre	164.47 (34.64) ^a^	168.55 (37.82)	0.307 (0.761) ^c^	235.991 (0.001)	0.013 (0.001)	88.311 (0.909)
	Post	179.22 (34.82)	172.11 (36.61)				
	Difference	−14.75 (3.12)	−3.55 (3.36)	9.397 (0.001)			
	95% CI	(−23.19 to 31.35)	(−23.03 to 31.19)				
	*t* (*p*)	−17.686 (0.001) ^b^	−4.219 (0.01)				
Hip abduction (*N*)	Pre	172.97 (59.30)	181.05 (59.96)	1.286 (0.209)	144.340 (0.001)	1.148 (0.293)	40.078 (0.001)
	Post	186.39 (59.88)	185.21 (60.15)				
	Difference	−13.41 (3.07)	−4.15 (4.65)	6.331 (0.001)			
	95% CI	(−16.64 to 72.80)	(−16.64 to 72.80)				
	*t* (*p*)	−16.336 (0.001)	−3.574 (0.003)				
External rotation (*N*)	Pre	113.00 (24.82)	128.78 (20.08)	1.924 (0.065)	132.975 (0.001)	1.997 (0.169)	46.073 (0.001)
	Post	124.11 (25.39)	131.66 (20.30)				
	Difference	−11.11 (3.97)	−2.87 (2.61)	6.788 (0.001)			
	95% CI	(−1.01 to 32.57)	(−1.35 to 32.91)				
	*t* (*p*)	−10.457 (0.001)	−4.410 (0.001)				

(*N*) = Newton; ^a^ M (SD); ^b^ paired *t*-test; ^c^ independent *t*-test; *F* = repeated measured ANOVA; HSE Group = hip-strengthening exercise group.

**Table 5:** Pre- and post-intervention static balance with eyes closed in hip strength exercise group and controls.

Parameters		HSE Group (*n* = 14)	Control Group (*n* = 16)	*t* (*p*)	Time *F* (*p*)	Group *F* (*p*)	Time × Group *F* (*p*)
CEA (mm^2^)	Pre	1150.40 (173.33) ^a^	1111.11 (221.01)	−0.536 (0.596) ^c^	155.619 (0.001)	0.167 (0.686)	0.352 (0.558)
	Post	918.37 (174.95)	900.15 (213.23)				
	Difference	232.02 (89.34)	210.96 (103.23)	−0.593 (0.558)			
	95% CI	(−189.43 to 110.85)	(−187.05 to 108.48)				
	*t* (*p*)	9.717 (0.001) ^b^	8.174 (0.001)				
PL (mm)	Pre	567.12 (65.98)	534.91 (81.52)	−1.178 (0.249)	292.231 (0.001)	0.069 (0.795)	7.564 (0.010)
	Post	388.59 (80.87)	405.80 (94.94)				
	Difference	178.52 (59.66)	129.11 (37.60)	−2.750 (0.010)			
	95% CI	(−88.21 to 23.80)	(−87.42 to 23.01)				
	*t* (*p*)	11.196 (0.001)	13.736 (0.001)				
AV (mm/s)	Pre	51.93 (12.89)	52.10 (16.99)	0.029 (0.977)	87.999 (0.001)	0.071 (0.792)	0.695 (0.411)
	Post	38.05 (6.64)	40.48 (15.51)				
	Difference	13.88 (8.16)	11.61 (6.72)	−0.834 (0.411)			
	95% CI	(−11.25 to 11.57)	(−11.04 to 11.37)				
	*t* (*p*)	6.361 (0.001)	6.912 (0.001)				

^a^ M (SD); ^b^ paired *t*-test; ^c^ independent *t*-test; *F* = repeated measured ANOVA; HSE group = hip-strengthening exercise group; CEA= 95% confidence ellipse area; PL = path length; AV = average velocity.

**Table 6:** Pre- and post-intervention dynamic balance in hip strength exercise group and controls.

Parameters		HSE Group (*n* = 14)	Control Group (*n* = 16)	*t* (*p*)	Time *F* (*p*)	Group *F* (*p*)	Time × Group *F* (*p*)
A (%)	Pre	65.83 (3.58) ^a^	63.26 (5.00)	−1.590 (0.123) ^c^	139.664 (0.001)	3.241 (0.083)	2.101 (0.158)
	Post	68.81 (3.96)	65.59 (4.82)				
	Difference	−2.98 (1.45)	−2.33 (0.98)	1.449 (0.158)			
	95% CI	(−5.86 to 0.73)	(−5.79 to 0.67)				
	*t* (*p*)	−7.649 (0.001) ^b^	−9.453 (0.001)				
P-L (%)	Pre	81.45 (5.19)	79.76 (6.74)	−1.325 (0.196)	286.910 (0.001)	4.387 (0.045)	19.331 (0.001)
	Post	92.10 (5.01)	85.94 (7.23)				
	Difference	−10.65 (2.19)	−6.18 (1.60)	6.445 (0.001)			
	95% CI	(−9.24 to 1.98)	(−9.14 to 1.88)				
	*t* (*p*)	−18.198 (0.001)	−15.449 (0.001)				
P-M (%)	Pre	83.74 (6.28)	80.11 (8.39)	−0.760 (0.453)	587.844 (0.001)	3.081 (0.090)	41.536 (0.001)
	Post	93.67 (5.18)	85.95 (9.10)				
	Difference	−9.92 (2.30)	−5.83 (2.73)	4.397 (0.001)			
	95% CI	(−6.24 to 2.86)	(−6.16 to 2.78)				
	*t* (*p*)	−16.141 (0.001)	−8.534 (0.001)				

^a^ M(SD); ^b^ paired *t*-test; ^c^ independent *t*-test; *F* = repeated measured ANOVA; HSE group = hip-strengthening exercise group; A = anterior; P-L = posterior–lateral; P-M = posterior–medial.

**Table 7:** Pre- and post-intervention results of FAAM in hip strength exercise group and controls.

Parameters		HSE Group (*n* = 14)	Control Group (*n* = 16)	*t* (*p*)	Time *F* (*p*)	Group *F* (*p*)	Time × Group *F* (*p*)
FAAM-ADL (%)	Pre	77.99 ± 6.41 ^a^	80.23 ± 8.20	0.823 (0.417) ^c^	207.714 (0.001)	0.016 (0.901)	8.581 (0.007)
	Post	89.61 ± 4.12	87.92 ± 5.22				
	Difference	−11.61 ± 2.97	−7.96 ± 4.16	2.929 (0.007)			
	95% CI	(−3.32 to 7.80)	(−3.24 to 7.71)				
	*t* (*p*)	−14.597 (0.001) ^b^	−14.058 (0.001)				
FAAM-SPORT (%)	Pre	64.73 ± 8.44	66.57 ± 7.54	−0.633 (0.532)	301.717 (0.001)	0.004 (0.949)	7.699 (0.010)
	Post	76.78 ± 8.37	75.30 ± 8.06				
	Difference	−12.05 ± 3.20	−8.73 ± 3.32	2.775 (0.010)			
	95% CI	(−4.13 to 7.82)	(−4.19 to 7.88)				
	*t* (*p*)	−7.390 (0.001)	−10.513 (0.001)				

^a^ M(SD); ^b^ paired *t*-test; ^c^ independent *t*-test; *F* = repeated-measures ANOVA; HSE group = hip-strengthening exercise group; FAAM-ADL = Foot and Ankle Ability Measure-ADL; FAAM-SPORT = Foot and Ankle Ability Measure-SPORT.

## Data Availability

Data are contained within the article.
